# Supplementary material for: Short-Term Effects of Different Types of Anti-Glaucoma Eyedrop on the Sclero-Conjunctival Vasculature Assessed Using Anterior Segment OCTA in Normal Human Eyes: A Pilot Study
Source: J Clin Med. 2020 Dec 11;9(12):4016. doi: 10.3390/jcm9124016 (PMC7764657; doi:10.3390/jcm9124016)
Supplement: Supplementary file 1 [file jcm-09-04016-s001.pdf]

Figure S1. AS-OCTA Image Acquisition and Processing.

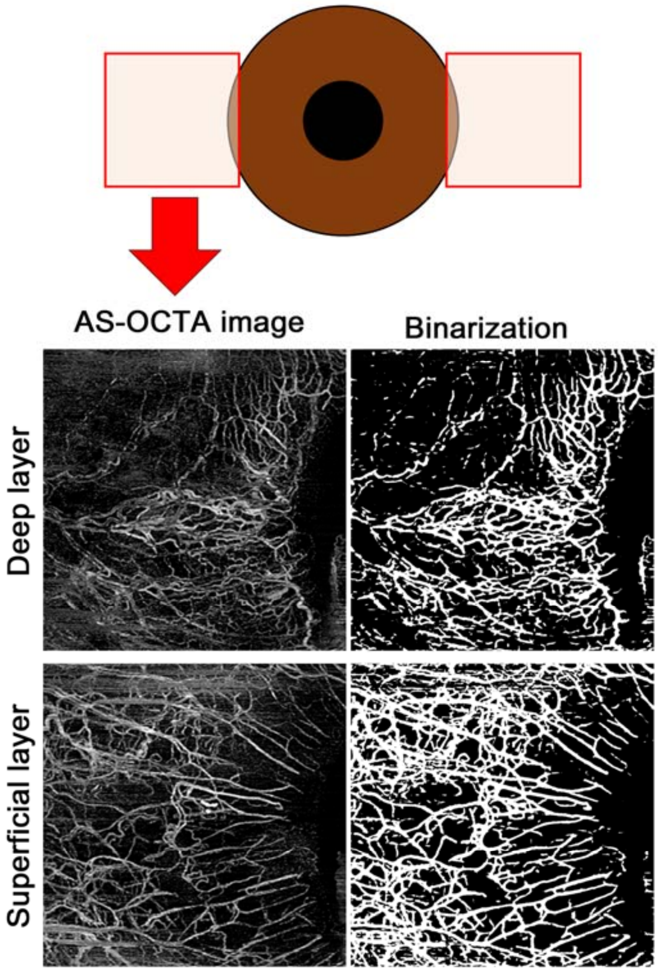

AS-OCTA images of the corneal limbus were obtained in the temporal and nasal regions. Binarization of each image in the deep and superficial layer was performed using a Trainable Weka Segmentation in ImageJ software.
